# Absence epilepsy might build its own nest

**DOI:** 10.1113/JP277642

**Published:** 2019-02-03

**Authors:** Gábor Kozák

**Affiliations:** ^1^ MTA‐SZTE ‘Lendület’ Oscillatory Neuronal Networks Research Group Department of Physiology University of Szeged Szeged H‐6720 Hungary

**Keywords:** absence epilepsy, Interneuron, epileptogenesis, electrophysiology

Absence epilepsy, the most common epilepsy of childhood, consists of brief seizures characterized by sudden, transient loss of consciousness, behavioural arrest and a spike‐and‐wave discharge (SWD)‐dominated EEG pattern, which can occur up to hundreds of times per day. It is widely accepted that in both human and genetic rodent models these SWDs are initiated in a hyperexcitable cortical focus and ictal activity is promptly generalized via corticocortical and thalamocortical connections. There are ample experimental demonstrations on the spiking dynamics of the major components of the thalamocortical loop during absence seizures. However, very little is known about the way cortical microcircuitry participates in seizure activity, and in particular the role of cortical interneurons remains to be elucidated. Furthermore, although the genotypic and phenotypic variations of absence epilepsy are well studied, still we have scarce knowledge on epileptogenesis in the seizure‐susceptible brain. Therefore pre‐existing epileptogenic conditions and seizure‐induced alterations of the thalamocortical circuitry have yet to be separated – if this is possible.

The study of Studer *et al*. (2019) in a recent issue of *The Journal of Physiology* shines new light on these questions. The authors performed a comparative anatomical, electrophysiological and behavioural study of genetic absence epilepsy rats from Strasbourg (GAERS) and non‐epileptic Wistar rats in order to identify possible alterations due to the recurrent seizures. First, based on the spectral and temporal characteristics of local field potentials of seizures, they precisely localized for the first time the seizure initiation zone inside the somatosensory cortex, which was found to be in the whisker region (S1Bf). Histological analysis of the putative seizure‐onset zone revealed that GAERS expressed an increased density of somatostatin positive (SST) and parvalbumin positive (PV) interneurons in layers 4 and 5 of the somatosensory cortex, respectively, compared to the non‐epileptic controls.

Studer *et al*. (2019) reported important structural differences between the epileptic and healthy rodent brain, but their study could not differentiate whether these alterations are contributors to the epileptogenesis or part of a compensatory mechanism which tries to dampen the effect of seizures on the cortical circuitry. It is known that during epileptogenesis SWDs progressively become more synchronous with a sharper, more pronounced spike component, reflecting that cortical units are firing more and more synchronously driven by the seizure activity (Jarre *et al*. 2017). Considering the temporal pattern of the PV cells’ activity (Pouille and Scanziani, 2004), the increased density of PV cells in layer 5 might account for the evolution of the SWD waveform during epileptogenesis, which can constrain the firing of excitatory cells into a narrow temporal windows within each SWD cycle, comparable to the width of the spike component of SWDs (∼20 ms; Jarre *et al*. 2017). The spectral profile of these sharp troughs can also serve as an explanation for the increased gamma power and SWD–gamma phase amplitude coupling during seizures, which was reported by the authors. Therefore, it is possible that the previously reported gradual change of SWD waveform parallels the increase of PV interneuron number in layer 5, and thus PV cells could be contributors to epileptogenesis.

An important aspect is whether these interneurons follow the same local connectivity rules as those in healthy animals. In the healthy brain, layer 4 SST neurons predominantly target local PV interneurons (Tremblay *et al*. 2016) and the activity of SST neurons is responsible for silencing other interneurons. Thus, via disinhibition, SST cells might strengthen the seizures instead of dampening ictal activity. In contrast, if one hypothesizes that PV cells actively contribute to epileptogenesis by fine‐tuning pyramidal cells during the spike components of SWDs, then the reported excess SST neuron pool in layer 4 could serve as a compensatory mechanism to dampen the reverberation of the thalamocortical loop in seizures. To judge if this is the case, it is critical to investigate the anatomical connectivity and firing dynamics of these interneurons during and between seizures.

In light of the differences of the cortical interneuron population, Studer *et al*. (2019) sought to investigate whether any functional deficiency of the epileptic cortex can be identified. They performed multi‐unit activity (MUA) and current source density (CSD) analysis on sensory evoked potentials induced by air‐puffs; then a texture discrimination test was performed to evaluate the behavioural correlates of the altered cortical microcircuitry. Interestingly, evoked potentials were delayed in GAERS and MUA revealed that cellular entrainment was also different as multi‐units were not only activated around the peak of the evoked potential as expected, but 300 ms later a secondary peak of firing was observed. It is somewhat surprising that no sign of this secondary activation was present on the CSD map of the evoked potentials. This clearly requires further investigations to reveal how such a massive concurrent activation of all the layers 2/3, 4 and 5/6 can occur without any obvious sinks or sources on the CSD map.

Despite the altered microstructure and evoked potential kinetics, no difference between GAERS and non‐epileptic Wistar rats in a texture discrimination test was detectable, and therefore the authors suggest that the sensory circuits of the somatosensory cortex are still able to process whisker‐related information. A recent study by Hong *et al*. (2018), which showed that even in the complete absence of barrel cortex mice are capable of performing whisker‐related tasks, provides a cautionary note. In light of this, it is possible that the methods used by Studer *et al*. (2019) might underestimate the functional impairment of somatosensory cortex, given that other brain regions are able to compensate for the missing or non‐functional somatosensory cortex.

The work of Studer *et al*. (2019) revealed important structural alterations of the epileptic brain of GAERS and highlighted that the altered structure of the somatosensory cortex might process sensory information in a substantially different way. Although absence epilepsy is considered a relatively benign form of epilepsy, it is often accompanied by comorbidities. In most cases patients become seizure‐free during late infancy, but the associated comorbidities might persist. Thus, their observations may have particular importance: the presented differences in the cortical interneuron populations might provide a background on which other neurophysiological impairments are more prone to occur. One caveat of the study of Studer *et al*. (2019) is that the applied methods did not make it possible to evaluate the function of inhibitory neurons or the dynamics of cortical inhibition. Multi‐unit activity analysis does not provide sufficient resolution to observe the temporal relationship between the firing of pyramidal cells and interneurons or between the different interneuron subpopulations. It would be beneficial to investigate sensory processing and seizure activity on a finer temporal and spatial scale. Simultaneous intra‐, juxta‐ or extracellular recordings of these cell types of the neocortex might provide an explanation of how these cells might contribute to or compensate the hyperexcitability of the seizure‐onset zone.

Furthermore, if the reported histological changes are at least partially due to compensatory mechanisms, it would be important to investigate whether the same interneuron density increase occurs in areas outside the seizure focus. This is a plausible assumption, since the seizures are generalized and all areas of the cortex are entrained to ictal activity to some extent. It is substantially different to locally compensate the aberrant ictal and interictal dynamics of the hyperexcitable core or to compensate seizure‐related widespread synchronous discharges of the cortex. In the latter case, this interneuron density increase in response to global seizures might have more serious consequences and could change the cortical computational mechanisms of the epileptic brain fundamentally.

The study of Studer *et al*. (2019) sets the stage for new hypotheses, although simultaneous unit recordings and a detailed connectivity analysis in all the cortical layers are required in the seizure onset zone and in other non‐sensory cortical areas. Only such experiments can further elucidate the impact of seizing on the cortical microcircuitry in absence epilepsy.

## Additional information

### Competing interests

None declared.

### Author contributions

Sole author.

### Funding

This work was supported by the 20391‐3/2018/FEKUSTRAT grant and the New National Excellence Program (UNKP‐18‐3‐III‐SZTE‐16) of the Ministry of Human Capacities, Hungary, and the ‘Momentum II’ Program of the Hungarian Academy of Sciences.
